# A Novel Two-Dimensional Liquid Chromatography Combined with Ultraviolet Detection Method for Quantitative Determination of Pyridoxal 5′-Phosphate, 4-Pyridoxine Acid and Pyridoxal in Animal Plasma

**DOI:** 10.3390/ani13081333

**Published:** 2023-04-13

**Authors:** Rong-Ju Yang, Na Wang, Xiao Ma, Meng-Die Gong, Yi-Rong Wang, Si-Yu Meng, Zhao-Ying Liu, Qi Tang

**Affiliations:** 1 College of Veterinary Medicine, Hunan Agricultural University, Changsha 410128, China; 2 Hunan Engineering Technology Research Center of Veterinary Drugs, Hunan Agricultural University, Changsha 410128, China; 3 College of Horticulture, Hunan Agricultural University, Changsha 410128, China

**Keywords:** two-dimensional liquid chromatography, vitamin B_6_, pyridoxal 5′-phosphate, 4-pyridoxic acid, pyridoxal

## Abstract

**Simple Summary:**

Vitamin B_6_ is directly or indirectly involved in many key biological metabolic processes in the body in the form of coenzyme factors, and it is able to maintain the normal progress of biological responses at very low levels, playing an important role in animal health and disease. In this study, a two-dimensional liquid chromatography-UV detector (2D-LC-UV) was used to establish a method for the simultaneous detection of PLP, PA, and PL for the first time. Its advantage of large volume injection provides adequate sensitivity. At the same time, multi-column is used to improve the shape of the peak, and the anti-interference ability is obviously improved. The system achieved rapid detection and stability quantification of PLP, PA, and PL. This method has been successfully applied to the plasma matrix of pigs, mice, and rats, providing a brand-new method for the determination of PLP, PA, and PL.

**Abstract:**

Vitamin B_6_ is an indispensable micronutrient in organisms and is widely distributed in blood, tissues, and organs. Changes in the content and ratio of vitamin B6 can affect the entire physiological condition of the body, so it becomes particularly important to reveal the relationship between changes in its content and disease by monitoring vitamin B6 levels in the organism. In this study, a two-dimensional liquid chromatography-UV detector (2D-LC-UV) was used to establish a method for the simultaneous detection of PLP, PA, and PL for the first time. First, PLP, PA, and PL were extracted with plasma: 0.6 M TCA: ultrapure water = 1:2:3 (*v*/*v*/*v*) and then derivatized. Enrichment and preliminary separation were performed on a one-dimensional column and automatically entered into a two-dimensional column for further separation. This method exhibited good selectivity, and the correlation coefficients for the analyte calibration curves were >0.99. The detection limits for PLP, PA, and PL were 0.1, 0.2, and 4 nmol/L, respectively. The results showed that the system has high loading capacity, excellent resolution, and a good peak shape. This method is expected to provide applicability for the determination of PLP, PA, and PL in pharmacological, pharmaceutical, and clinical research.

## 1. Introduction

Vitamin B_6_ participates in more than 100 enzymatic reactions as a coenzyme, including decarboxylation, racemization, transamination, elimination, and aldol cleavage, which play different functions in amino acid, sugar, and fat metabolism [1,2,3] and constitute the necessary conditions for neurotransmitter biosynthesis by differentiation, proliferation, migration, and maturation of neurons [4]. Vitamin B6 plays a vital role in biological processes that include protecting nerves, enhancing immunity [5], and preventing cancer [6]. Vitamin B_6_ deficiency has a significant impact on physiological functions, increasing the risk of cardiovascular disease, renal dysfunction, cancer, chronic inflammation, and other diseases [7,8,9,10,11] and decreasing weight, growth velocity, and blood glucose regulation ability [12,13,14].At present, it has not been determined which vitamin B_6_ should be used as an indicator to evaluate the whole state, but pyridoxal 5-phosphate (PLP), 4-pyridoxine acid (PA), and pyridoxal (PL) have been proposed. Plasma PLP is generally used to evaluate the overall vitamin B_6_ status due to its highest content [15,16]. PL is the highest content of vitamin B_6_ in the brain and is used to assess the risk of vitamin B_6_-responsive epilepsy with PLP. PA is an uncombined metabolite of vitamin B_6_ that is a potential multifunctional marker to reflect recent vitamin B_6_ status with easier detection [17]. Therefore, a simultaneous measurement method of PLP, PL, and PA, which is more objective, reasonable, and comprehensive, would be helpful in exploring the relationship among vitamin B_6_ levels, disease mechanisms, and body health.

Detecting vitamin B_6_ methods have been developed since the 1950s, such as microbial methods [18], enzymatic processes [19,20], high-performance liquid chromatography (HPLC) [21,22,23], liquid chromatography mass-spectrometry (LC-MS) [24,25,26,27,28], etc. Early microbial and enzymatic processes were gradually eliminated due to preprocessing steps, high throughput, sensitivity, universality, and other reasons. In recent years, due to the wide selection of analytical columns and detectors, HPLC has become the main method for the determination of vitamin B_6_ [21,22,29,30,31]. Fluorescence detection (FLD) is more sensitive than UV and more suitable for trace analysis. In many cases, the high selectivity of FLD avoids interference by nonfluorescent components. PA can naturally produce fluorescence; PLP and PL can be induced by complex precolumn or post column derivatization reaction fluorescence, which is detected by FLD; while derivatization reactions can enhance the fluorescence intensity of PA. Because the relationship between detection wavelength ranges is more sensitive than UV detection, FLD also has a wider quantitative range for vitamin B_6_. LC-MS reflects the complementary advantages of chromatography and mass spectrometry and integrates the strong separation ability of high-performance liquid chromatography for complex samples with the high specificity, high sensitivity, and high selectivity of mass spectrometry. However, there are few methods to detect vitamin B6 in biological samples using LC-MS/MS alone [25,26,28], mostly simultaneously with other vitamins or metabolites [32,33], and the versatility of the method is good, but the specificity of the method is not strong relative to vitamin B_6_ compounds.

Two-dimensional liquid chromatography (2D-LC) is a system in which two columns with independent separation mechanisms are combined to achieve complex sample separation by changing multiple mobile phases through a valve switching device [34,35]. Samples are first extracted and enriched, and impurities are removed by the first-dimension column, and then the sample is entered into the second-dimension column for further separation. Orthogonality and peak capacity are important parameters to evaluate the separation ability of 2D-LC in 2D-LC systems. The better the orthogonality, the better the separation ability of 2D-LC. The greatest feature of 2D-LC is the effective reduction in overlap between components, thereby increasing peak capacity. As a chromatographic mode emerging in recent years, 2D-LC compensates for the shortcomings of weak injection ability, poor separation ability, and low sensitivity of traditional one-dimensional chromatography (1D-LC), and it effectively improves the separation ability, resolution, sensitivity, and system peak capacity and reduces impurity interference of complex samples [36,37]. Therefore, a two-dimensional liquid phase method for simultaneous detection of PLP, PA, and PL in animal plasma was established, and the method was investigated and applied in practice.

## 2. Materials and Methods

### 2.1. Chemicals and Reagents

Pyridoxal 5′-phosphate (CAS 853645-22-4, molecular formula C_8_H_10_NO_6_P·xH_2_O, molecular weight 247.14, purity ≥ 98.0%), 4-pyridoxic acid (CAS 82-82-6, molecular formula C_8_H_9_NO_4_, molecular weight 183.16, purity ≥ 98.0%), pyridoxal hydrochloride (CAS 65-22-5, molecular formula C_8_H_9_NO_3_·HCl, molecular weight 203.62, purity ≥ 99.0%), and semicarbazide hydrochloride (CAS 563-41-7, molecular formula NH_2_CONHNH_2_·HCl, molecular weight 111.53, purity ≥ 99.0%) were purchased from Sigma Aldrich (St Louis, MO, USA). Glycine was purchased from Beijing Solar Science & Technology Co., Ltd. (Beijing, China). Ammonium dihydrogen phosphate was obtained from Sinopharm Chemical Reagent Co., Ltd. (Shanghai, China). Trichloroacetic acid was obtained from Tianjin Cameo Chemical Reagent Co., Ltd. (Tianjin, China). Acetonitrile and methanol (HPLC grade) were obtained from Merck Co. (Darmstadt, Germany). All other reagents used were of optimal analytical quality. Pure water was purified using a Milli-Q purification system (Millipore, Bedford, MA, USA).

### 2.2. Plasma Samples

Blank mouse and rat plasma samples were collected from Hunan SJA Laboratory Animal Co., Ltd. (Changsha, China). The animal experiments were approved by the Ethics Committee of Hunan Agricultural University (Number: 2020-43). Blank pig plasma samples were donated by Hunan New Wufeng Co., Ltd. (Liuyang, China).

### 2.3. Instruments and 2D-LC Conditions

The equipment and chromatographic conditions were tested by a Shimadzu and ANAX combined system. The FLC-2420 column oven of the 2D-LC system was from ANAX (Changsha, China). Three high-pressure pumps (Pump A is a high-pressure gradient chromatography LC-20AT pump; Pump B and Pump C are high-pressure LC-20AT pumps), an SPD-20A UV detector, a SIL-20A auto-sampler (with a 600-μL injection loop), a CBM-20A system controller, and three six-port two-position FCV-12AH switching valves of the 2D-LC system are all from Shimadzu (Kyoto, Japan).

The first-dimensional liquid chromatogram (1D) LC separation used an SPY column (50 mm × 4.6 mm, 5 μm, Changsha, China; LC1). The mobile phase used for Pump C was methanol and 0.02 mol/L ammonium acetate (pH = 6.1) (5:95, *v*/*v*), with a flow rate of 1.0 mL/min. The second-dimensional (2D) LC separation used a C18 column (Waters Summetry^®^, 4.6 × 150 mm, 5 µm; LC2). The mobile phase used for Pump A was methanol, acetonitrile, and 0.02 mol/L ammonium acetate (pH = 6.1) (9:6:85, *v*/*v*/*v*), with a flow rate of 1.0 mL/min. One-quarter of 0.02 mol/L ammonium dihydrogen phosphate (20 mM) was added to ammonium acetate to improve the chromatographic peak pattern. The chromatographic separations were performed in the isometric elution. The UV response of PLP and PL after derivatization was enhanced, and the maximum UV absorption wavelength was changed. Therefore, it was feasible to change the absorption wavelength of PLP and PL through the derivatization reaction to increase the response value to improve the sensitivity. For general considerations, it was determined that 310 nm was the UV detection wavelength of the three vitamin B_6_ samples. The separation process was set up at 40 °C. PLP, PA, and PL were enriched online on a one-dimensional column separated in the first dimension and then entered into a second-dimensional column for further separation and detection. The two-dimensional systems were connected via an Intelligent Fluid Path Control System (TRS) using a “center cut” to transfer the target. The transfer time procedures for PLP, PA, and PL are shown in Table 1.

### 2.4. Standard Solutions, Calibration Curves and Quality Control Samples

Standard stock solutions of PLP and PL were dissolved in a small volume of 1 mol/L HCl and diluted to 100 mmol/L in ultrapure water, respectively. PA standard stock solution was dissolved in 1 mol/L NaOH and diluted to 10 mmol/L in ultrapure water. Stock solutions were transferred to brown bottles and frozen at −80 °C.

The calibration curves were obtained by plotting the peak areas of PLP, PA, and PL against their corresponding concentrations. All samples, including the calibrated samples and quality control samples (QCs) at all levels, were divided into aliquots and placed in Eppendorf tubes (2.0 mL), stored at −80 °C, and brought to room temperature before use.

To prepare calibration curves, known amounts of working solutions at nine concentrations for PLP, PA, and PL (0.2, 0.6, 60, 100, 300, 500, 1000, 2000, and 10,000 nmol/L, respectively) were added to the plasma samples. The blank plasma samples of pig, mice, and rats were extracted and treated, and then the standard work was diluted into quality control solutions of different concentrations after 50 times dilution in ultra-pure water. For QCs, four concentration levels were used (PLP: 0.2, 0.6, 60, and 250 nmol/L; PA: 0.4, 1.2, 100, and 300 nmol/L; PL: 20, 60, 1000, and 1500 nmol/L).

### 2.5. Sample Pretreatment

Blood was collected from the hearts of rats and mice after anesthesia. The blood was placed in anticoagulant tubes and centrifuged at 4 °C at 3000 rpm for 15 min immediately. The supernatant plasma was separated in brown centrifuge tubes. The collected porcine anterior venous blood was placed in an anticoagulant tube and centrifuged at 3000 rpm at 4 °C for 15 min immediately, and the supernatant plasma was collected in a brown centrifuge tube, all of which was frozen at −80 °C.

#### 2.5.1. The Extraction Procedure

Sixty microliters of fresh plasma from pigs, mice, and rats were transferred into a 2-mL brown centrifuge tube; 120 μL of 0.6 M trichloroacetic acid solution was added, vortexed for 30 s, diluted with 180 μL of ultrapure water, mixed well, allowed to stand at 50 °C for 5 min, and centrifuged at 10,000 rpm at 4 °C for 10 min; and the supernatant was collected.

#### 2.5.2. The Derived Procedure

Three hundred microliters of mixed standard solution or blank plasma supernatant was transferred into a 1.5-mL brown centrifuge tube, mixed well with 350 μL semicarbazide solution at 10 mmol/L, reacted in a constant temperature shaker at 50 °C for 20 min at 300 rpm in the dark, passed through a 0.22-μm filter membrane, and tested by a well-balanced 2D-LC system.

### 2.6. 2D-LC Capability Assessment

#### 2.6.1. 2D-LC Online Processing Capability Assessment

Online processing capability assessment of 2D-LC was performed experimentally to avoid shifts and distortions of the chromatographic peaks due to increasing injected volumes. Using a mixed standard solution containing 10 μmol/L, after pretreatment, 10, 20, 50, 100, 200, and 500 μL were injected according to the chromatographic conditions, and the peak area (As) values of PLP, PA, and PL were used as the dependent variable (Y). The sample quantity (Am) was the independent variable (X), and the regression curve was fitted by the least squares method to obtain a fitting equation.

#### 2.6.2. 2D-LC Transfer Capability Assessment

To realize the transfer of PLP, PA, and PL from the one-dimensional column to the two-dimensional column, the degree of transfer of the three targets is a key indicator for assessing the transfer performance of the 2D-LC system; that is, the transfer recovery and transfer precision are calculated by the PLP, PA, and PL peak areas under 2D-LC conditions and LC2 conditions, and the transfer recovery (Rtr) is expressed by the ratio of the two; the transfer precision is expressed by the coefficient of variation (CV%) among the PLP, PA, and PL peak areas obtained multiple times under 2D-LC conditions.

After pretreatment of the mixed standard solutions of PLP, PA, and PL with different concentrations, the samples were injected under LC2 and 2D-LC conditions. Each concentration was repeated three times, and the transfer recovery (Rtr) and transfer precision (CV%) of PLP, PA, and PL were calculated.

### 2.7. Method Validation Procedure

The method validation was performed in accordance with the U.S. Bioanalytical Method Validation Guidelines [38]. In the process of analysis and verification, we tested the selectivity, sensitivity (limit of detection (LOD) and limit of quantification (LOQ)), linearity, precision (intra-and interassay variation), accuracy, recovery, and stability of PLP, PA, and PL in vitamin B_6_ compounds.

Six blank samples were selected from plasma biological samples of pigs, mice, and rats for analysis. Compared with the injection analysis of the alternative matrix (Milli-Q water), their interference in the retention time and selectivity of PLP, PA, and PL were evaluated.

LOD and LOQ are defined as the lowest concentrations in the matrix that can be detected and quantified. Their signal-to-noise ratios (S/N) were ≥3.3 and 10, respectively. A standard curve was established using LOQ as the starting concentration point, the injected concentration (C) as the independent variable (X), and the peak area (As) as the dependent variable (Y), and the regression curve was fitted according to the least squares method with R^2^ > 0.99 to obtain the fitting equations for PLP, PA, and PL.

The precision and accuracy were determined by analyzing QC solutions. Plasma LLOQ, LQC, MQC, and HQC solutions of pigs, mice, and rats were pretreated and injected according to 2D-LC conditions. Each concentration sample was measured five times for three consecutive days, and intra-day and inter-day precision, accuracy, and recovery rates of PLP, PA, and PL were calculated.

Pig, mouse, and rat plasma QCL, QCM, and QCH were stored in a 4 °C for three days and six replicates of each concentration; after pretreatment, samples were injected according to 2D-LC chromatographic conditions. Short-term stability of QCs in 4 °C was verified.

### 2.8. Application of the Method

In this experiment, the calibration curves of pig, mouse, and rat plasma samples were established for simultaneous quantitative analysis of PLP, PA, and PL, and the method was studied to prove the applicability of the method.

## 3. Results

### 3.1. Pretreatment Condition Results

#### 3.1.1. Extraction Condition Results

**Figure 1:**
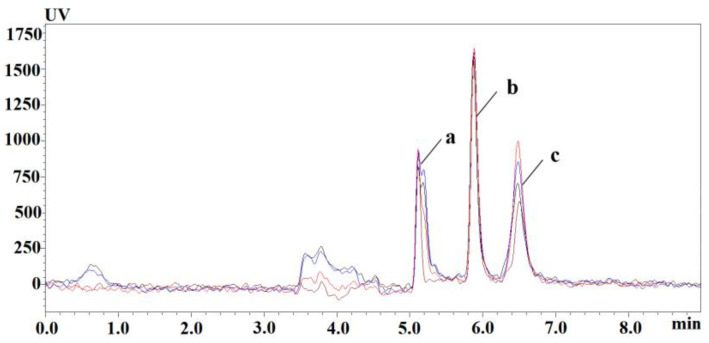
Chromatograms of plasma PLP, PA, and PL extracted by TCA at different concentrations: red-0.6 M TCA; blue-5% TCA; black-10% TCA; brown-reference substance. (a) PLP, (b) PA, (c) PL.

The preparation of vitamin B6 sample requires extraction and purification steps to reduce the pollution by impurities of the equipment and to ensure the precision and stability of the analysis method. Under the same pretreatment conditions, the recovery of trichloroacetic acid and organic reagent-treated samples was investigated. As shown in Figure 1, 0.6 M TCA had good recovery; the extraction recoveries of different organic reagents are shown in Table 2; the extraction yields of PLP were generally low; and the extraction recoveries of PLP, PA, and PA were relatively good at methanol: acetonitrile = 6:4 (*v*/*v*). Trichloroacetic acid (0.6 M) was selected as a precipitating protein reagent to further optimize the extraction conditions of vitamin B_6_.

Under the same conditions, 50 μL of mouse plasma were used to investigate the addition volume of TCA and the dilution ratio of ultrapure water, as shown in Table 3. Plasma was extracted with more than two volumes of 0.6 M TCA, and the recoveries of PLP, PA, and PL were good; the plasma was diluted without water, or the dilution volume was less than 50 μL (plasma: 0.6 M TCA: water = 1:2:1, *v*/*v*/*v*); the recoveries of PLP and PL were low; the recoveries of PLP were higher than 120% at the dilution volume of 100 μL; the recoveries of PL were within the normal range, and the PA chromatographic peak was deformed; and the recoveries were greater than 120%; considering the above results, extraction was performed according to plasma:0.6 M TCA: ultrapure water = 1:2:3 (*v*/*v*/*v*).

Incubation times and temperatures [39,40,41] were investigated under established extraction conditions, as shown in Table S1. PLP, PA, and PL showed good recoveries under different incubation conditions; the longer the incubation time, the lower the recoveries of analytes; and the incubation condition of 50 °C for 5 min was selected considering the rapidity and efficiency of pretreatment.

#### 3.1.2. Derived Condition Results

**Figure 2:**
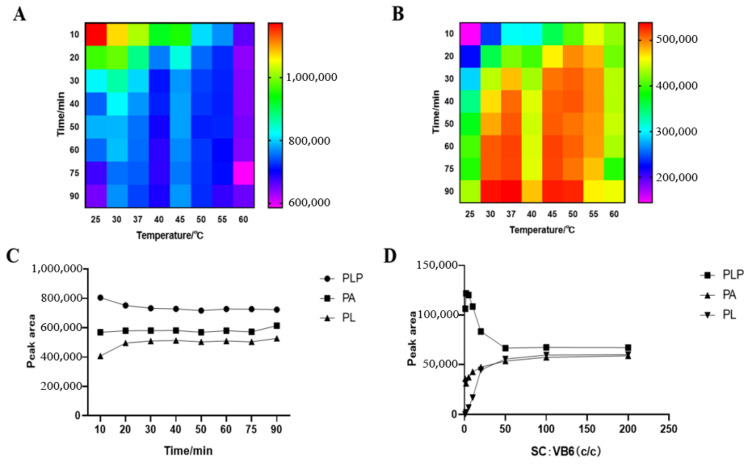
Derivatization conditions of PLP and PL: (**A**) PLP; (**B**) PL; (**C**) Derivative reactions of PLP, PA and PL in different times at 50 °C, (**D**) Influence of semicarbazide concentration on derivatization reaction.

We studied the influence of semicarbazide concentration on the derivatization reaction and the optimum time and temperature of the derivatization reaction. The result is shown in Figure 2. The shorter the PLP reaction time, the higher the response, the higher the reaction temperature, and the faster the reaction tends to be stable; the lower the PL temperature, the lower the response, and there is a higher response value between 30 and 50 °C. For the purpose of rapid reaction, 50 °C was selected to react in the dark for 20 min. The mixed standard reacted completely with semicarbazide at a concentration ratio of 1:50, and PLP and PL in the sample could react completely with semicarbazide when the concentration of semicarbazide exceeded 50 times that of the analyte, while the derivatization reaction of some PA with semicarbazide could also be seen by the trend of PA.

### 3.2. 2D-LCsystem Performance Analysis Results

#### 3.2.1. 2D-LC System Online Processing Capability Results

The loading capacity of the 2D-LC column is up to 600 μL, while that of a conventional liquid chromatography column usually does not exceed 50 μL, so the separation capacity and sensitivity of the system can be improved. After pretreatment, 10, 50, 100, 200, 300, and 400 μL of the added samples at 10 μmol/L were injected; PLP, PA, and PL peak areas (As) were used as dependent variables, and injection volume (Am) was used as the independent variable (X) to establish a linear relationship between injection volume and peak areas; and the regression curve was fitted according to the least squares method to obtain the fitting equation Table 4.

**Figure 3:**
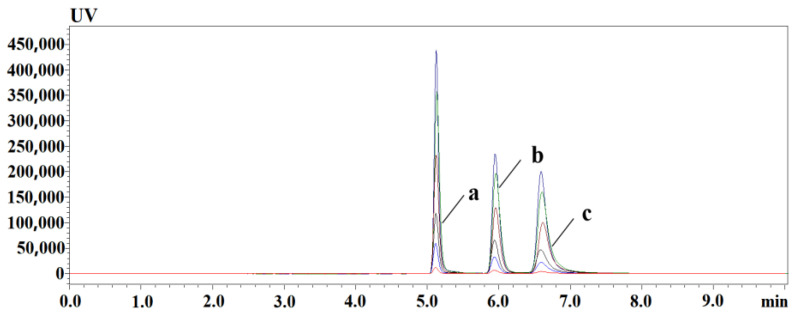
PLP, PA, and PL injection volume versus response; online enrichment chromatogram of different injection volume, from bottom to top; the injection volume is 10~400 μL. (a) PLP; (b) PA; (c) PL.

The results show that there is a good linear relationship between the injection volume and the peak area in the range of 10 to 400 μL, and R^2^ is greater than 0.99. Large volume injection did not cause linear distortion of PLP, PA, and PL. The chromatographic peaks of PLP, PA, and PL were not distorted or shifted, showing that this two-dimensional liquid chromatography has good on-line enrichment ability for PLP, PA, and PL in samples, as shown in Figure 3.

#### 3.2.2. 2D-LC Transfer Capability Assay Results

The transfer capability of this system was assessed with transfer recovery (Rtr) and transfer precision (CV%) of samples at different concentrations, as shown in Table 5. The average transfer recoveries of PLP, PA, and PL at low, medium, and high concentrations were more than 90%, demonstrating complete transfer from the extraction column to the analytical column during column-to-column switching. The CV% of PLP, PA, and PL at different concentrations were within 8.9%, and the transfer stability of the 2D-LC system was good.

### 3.3. Method Validation Results

#### 3.3.1. Selectivity

**Figure 4:**
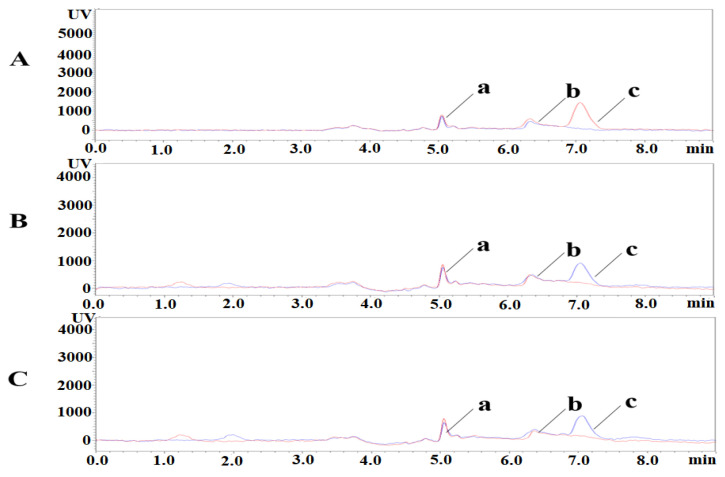
Specificity chromatograms of PLP, PA, and PL. a: PLP; b: PA; c: PL. (**A**) Pig, blue-blank sample, red-added sample; (**B**) Mouse, red-blank sample, blue-added sample; (**C**) Rat, red-blank sample, blue-added sample. (a) PLP; (b) PA; (c) PL.

PLP, PA, and PL were isolated under the liquid phase conditions described above. The retention times of PLP, PA, and PL were 5.1 min, 6.3 min, and 7.1 min, respectively. Representative chromatograms of blank plasma samples and spiked low concentration samples are shown in Figure 4. The retention time and peak profile of PLP, PA, and PL peaks did not change in the added samples. The method has good selectivity and is not influenced by endogenous substances in biological matrices.

#### 3.3.2. LOD, LOQ, and Linearity

The results of LOD and LOQ for PLP, PA, and PL are shown in Table 6. The LOD of PLP, PA, and PL was 0.1, 0.2, and 4 nmol/L, respectively, and the LOQ of PLP, PA, and PL was 0.2, 0.4, and 20 nmol/L, respectively, in the standard curve based on ultra-pure water. Using water as the matrix to make standard curves, PLP, PA, and PL had an excellent linear relationship within the quantitative range, R^2^ > 0.99, repeated three times, and the RSD% was within 4%. The LOD and LOQ of the matrix-added samples were the same as those of the standard, which reached the same sensitivity as the existing HPLC-FLD and LC-MS methods [15,25,42,43,44,45].

When generating the matrix standard curves, to reduce endogenous interference, the plasma matrix was diluted 50 times and 100 times with ultrapure water to explore suitable treatment methods. The test results are shown in Table S2. By comparing the relative standard deviations (RSDs) of plasma matrix curves and standard curves, plasma was diluted 50 and 100 times. Plasma diluted 50 times had less endogenous interference, and the plasma matrix curve was closer to the standard curves (RSD% < 15%), with remarkable parallelism and the linear relationship (R^2^ > 0.99) as the method verification object. The results proved that ultrapure water could not be used as an equivalent substitute for the plasma matrix, and water substitute matrix also led to high matrix effects and analytical inaccuracies in LC-MS/MS [43].

#### 3.3.3. Precision, Accuracy and Recovery

Four levels with replicates (*n* = 5) were analyzed for intra-day and inter-day quality control assays. The results are shown in Table 7. The intra-day precision values were <12.6%. The accuracy varied from −7.61% to 15.38%. For the acceptance criteria for precision and accuracy, the results should conform to a coefficient of variation (CV) of ≤15% except for a coefficient of variation of LLOQ ≤ 20. The recovery values ranged from 90.00% to 113.90% with variability (CV) of <12.51%. The results showed that the method can be used for the quantitative analysis of PLP, PA, and PL in pig, mouse, and rat plasma.

#### 3.3.4. Stability

The stability of the method was expressed as relative standard deviation (RSD%). Table 8 shows the short-term stability results of pig, mouse, and rat plasma QCL, QCM and QCH samples after being placed at 4 °C for three days. The RSD% of pig plasma was 2.3% to 13.66%, mouse plasma was 2.6% to 13.37%, and rat plasma was 2.11% to 14.97%, suggested that the samples were placed at 4 °C for three days without analyte degradation, and the stability was good.

### 3.4. Method Application Results

A total of more than 30 valid samples were collected, and 2D-LC detection was performed after pre-processing. The results are shown in Table 9. Pig plasma PLP was 197~431 nmol/L, that of PL was 82~185 nmol/L, and that of PA was 3~15 nmol/L. Mouse plasma PLP was 131~256 nmol/L, that of PL was 25~130 nmol/L, and that of PA was10~15 nmol/L. Rat plasma PLP was 151~300 nmol/L, that of PA was 9~51 nmol/L, and that of PL was 21~36 nmol/L.

## 4. Discussion

For separating PLP, PA, and PL from endogenous substances, the stationary phase was selected for reversed-phase C18 and C8 column [29,46,47,48,49,50,51], and the mobile phase was isocratic elution, which requires a suitable buffer added to adjust the pH and the elimination of overlapping peaks, tail peaks, and shoulder peaks. A Waters Summetry^®^ C18 column was selected as the 2D column, and the Aston TM^®^ SPY column (phenyl hexyl column, Hunan Demite instrument Co., Ltd., Changsha, China) was chosen as the 1D column, which had simultaneous retention of three analytes, and the mobile phases of the two dimensions had to be compatible. Acidic pH reduces the polarity of PLP and promotes its retention on the chromatographic column. Phosphate buffer and methanol were used as the original mobile phase. However, the pH of the phosphate buffer was unstable and easily crystallized when meeting methanol, and it blocked the internal pipeline, so the water phase was changed to a more stable ammonium acetate solution. In the 20 mM ammonium acetate solution with pH = 6.8, PLP, PA, and PL had the best resolution, but the PL peak was severely tailed. After adding acetonitrile and ammonium dihydrogen phosphate solution to the mobile phase, the PL peak tailing factor decreased, and the three compounds had good separation. The time running program is directly related to the transfer ability of the target from the extraction column to the analysis column and the sample load of the system; that is, the time window is too wide, the PLP peak loss is serious, the analyte sample load is small, the time window is too narrow, and the PL transfer is incomplete. Through the results of transfer recovery rates in different time windows, it was found that the target substance began to elute at 2.0 min and completely eluted at 3.5 min.

Vitamin B_6_ binds tightly to proteins and must release the analyte from the protein under the premise of ensuring that the analyte in the sample is not degraded or metabolized. Precipitated protein method is a feasible sample preparation technique. Protein precipitants include trichloroacetic acid [52,53], perchloric acid [21,50,54], metaphosphoric acid [55], formic acid [56], methanol, acetonitrile, and so on. Among them, trichloroacetic acid and perchloric acid are most useful for releasing vitamin B_6_. Perchloric acid has strong acidity, which exceeds the acid–base tolerance range of the column. It often requires alkaline reagents to adjust the pH of the sample solution to protect the column, such as NaOH, KOH, and NH_4_OH, which were among many uncontrollable factors in the sample pretreatment. In contrast, trichloroacetic acid, methanol, and acetonitrile are ideal extraction reagents and were investigated. According to previous studies [39,40], the extraction reagent, dilution ratio, and incubation conditions of the extraction reagents were investigated. We found that, when plasma:0.6 M TCA: ultrapure water = 1:2:3 (*v*/*v*/*v*) and is incubated at 50 °C for 5 min, the extraction recovery rate was good.

The structural characteristics of PLP and PL determine that they do not have strong ultraviolet absorption and fluorescence. Through derivatization reactions, the analytes obtained or enhanced ultraviolet absorption and fluorescence characteristics, thereby improving the sensitivity and specificity. Zhang [57] investigated the effect of various chlorites, bisulfites, semicarbazide derivatization reagents, and methods on PLP derivatization. Sodium bisulfite precolumn derivatization obtained the highest signal-to-noise ratio but a short retention time. In contrast, the pre-column derivatization of semicarbazide produced a higher signal-to-noise ratio and a more acceptable on-column retention time. Considering the safety of derivatization reagents and the stability of derivatized products, the semicarbazide pre-column derivatization method is a more suitable derivatization method. In this study, we investigated the effects of the optimal time, temperature, and semicarbazide concentration on the semicarbazide-derivatization reaction. Semicarbazide requires more than a 50-fold concentration to fully derive with PLP and PL, and some PA is also reacted. The derivatization of the target reacts for 5 min at 50 °C in the dark, consistent with the extraction temperature, and TCA extraction and derivatization can be synchronized, followed by centrifugation to obtain the supernatant to reduce the depletion of the target during the extraction process.

HPLC detection methods have been relatively mature and easy to perform, with accurate determination and good repeatability. Fluorescence detection generally has good specificity and sensitivity. There are many studies of PLP, PA, and PL based on fluorescence detection, and a universal set of detection methods has been basically formed [29,30,31]. In this study, the LOQ of PLP was 0.2 nmol/L, that of PA was 0.4 nmol/L, and that of PL was 20 nmol/L. This result is similar to that obtained by other authors when quantifying vitamin B_6_ samples, in which PLP is 3.5 to 4.5 nmol/L, PA is 1.2 to 6.3 nmol/L, and PL is 2.0 to 20 nmol/L [51,58]. Van der Ham M’s quantitative limit for the determination of PLP in human cerebrospinal fluid by ultra-performance liquid chromatography-mass spectrometry is 0.38 nmol/L, as well as 0.09 nmol/L for PA and 1.54 nmol/L for PL [26]. It must be acknowledged that high performance liquid chromatography-mass spectrometry (HPLC/MS) methods have higher sensitivity than high performance liquid chromatography-ultraviolet and high-performance liquid chromatography-fluorescence detection (HPLC-FL) methods. The HPLC-MS/MS method for vitamin B_6_ analysis has certain advantages, but HPLC-MS/MS detection equipment is expensive, it is difficult to operate the instrument, there are high maintenance costs, and it is not suitable for factory and laboratory routine application. Therefore, we have established a 2D liquid chromatography method for the simultaneous detection of PLP, PA, and PL in animal plasma, which provides more choices for the accurate quantification of PLP, PA, and PL. We also applied this method to animal plasma samples and determined the contents of PLP, PA, and PL in the plasma of pigs, mice, and rats. Due to the different ages of experimental pigs, the plasma PLP level of pigs has a large fluctuation range and is higher than the previous level. The plasma PLP and PL levels of mice are much higher than the reference values, which might be caused by not fasting them before sampling. The plasma PLP of rats is 151~300 nmol/L, that of PA is 9~51 nmol/L, and that of PL is 21~36 nmol/L, all of which are normal concentrations. This method has good applicability and accuracy.

## 5. Conclusions

In conclusion, we established for the first time a rapid, automatic, and low-cost 2D-LC method for simultaneous quantification of PLP, PA, and PL in animal plasma. The system provides a large-capacity injection with good detection sensitivity and strong anti-interference ability. After a short period of incubation at a high temperature, trichloroacetic acid releases plasma PLP, precipitates protein, and removes impurities in the pretreatment process. To improve the sensitivity of ultraviolet detection, PLP, PA, and PL were derivatized. The method has a low detection limit and LOQ and good specificity, precision, accuracy and short-term stability. This method has been successfully applied to plasma samples of pigs, mice, and rats, providing a new method for clinical trials and achieving the target of large-scale, low-cost, and wide-ranging sample detection.

## Figures and Tables

**Table 1:** Time program for transferring PLP, PA, and PL.

Time	Command	Major Function
0.00 min	1D column is disconnected from 2D column	The compound enters the 1D column for preliminary separation
2.00 min	1D column is disconnected from 2D column	The compounds are transferred from the 1D column to the 2D column for further separation
3.50 min	1D column is disconnected from 2D column	The compounds are completely eluted from the 2D column and enters UV detection
8.5 min	Stop	End

**Table 2:** Extraction recoveries of organic reagents in different proportions.

Volume Ratio	Compounds			Volume Ratio	Compounds		
	PLP	PA	PL		PLP	PA	PL
Met:Ace (2:8)	71%	87%	97%	Met:Ace (4:6)	79%	91%	85%
Met:Ace (6:4)	85%	95%	92%	Met:Ace (8:2)	77%	89%	111%
Met:W (2:8)	77%	83%	113%	Met:W (4:6)	76%	85%	99%
Met:W (6:4)	71%	83%	102%	Met:W (8:2)	72%	88%	101%
Ace:W (2:8)	73%	88%	118%	Ace:W (4:6)	66%	89%	99%
Ace:W (6:4)	63%	98%	117%	Ace:W (8:2)	63%	91%	112%

Note: “Met” means methanol; “Ace” means acetonitrile; “W” means water.

**Table 3:** Extraction recovery with different volumes of extraction reagent and ultrapure water.

Compound	Added Volume						Added Ultrapure Water					
	50 μL		100 μL		200 μL		0 μL	50 μL	100 μL	150 μL	200 μL	300 μL
	TCA (2%)	TCA (0.6 M)	TCA (2%)	TCA (0.6 M)	TCA (2%)	TCA (0.6 M)						
PLP	90%	89%	100%	96%	80%	96%	69%	69%	164%	128%	95%	96%
PA	156%	123%	108%	108%	80%	108%	129%	125%	121%	127%	135%	108%
PL	184%	126%	131%	108%	109%	108%	65%	60%	117%	113%	98%	108%

**Table 4:** Online processing capability assessment form for PLP, PA, and PL.

Compound	Linearity, Range	R^2^
PLP	Y = 5683.4x + 36,694, 10~400 μL	0.9956
PA	Y = 4843.4x + 11,969, 10~400 μL	0.9977
PL	Y = 6179x + 27,586, 10~400 μL	0.9965

**Table 5:** Online processing capability assessment form for PLP, PA, and PL transfer recovery rate (Rtr%) and transfer precision (CV%) of 2D-LC system.

**Concentration (μmol/L)**	**PLP**		**PA**		**PL**	
	Rtr%	CV%	Rtr%	CV%	Rtr%	CV%
10	117%	2.3%	98%	0.8%	90%	8.9%
50	98%	3.7%	100%	1.5%	92%	7.4%
100	96%	5.5%	101%	1.8%	93%	6.9%

**Table 6:** LOD, LOQ, and linear relationship of PLP, PA, and PL.

Compounds	Linearity	Linear Range (nmol/L)	Slope	R^2^	LOD	LOQ
PLP	Y = 325.15X + 2942.8	0.2~400	3.48%	0.9997	0.1	0.2
PA	Y = 251.94X + 22989.6	0.4~400	3.89%	0.9999	0.2	0.4
PL	Y = 307.07X + 10257	20~2000	3.87%	0.9944	4	20

**Table 7:** Intra-and inter-day precision (CV), accuracy (bias), and recovery of PLP, PA, and PL in plasma of pigs, mouse and rats.

Animal	Compound	QC Samples (*n* = 5)	CV (%)		Bias (%)	Recovery (%)	
			Intra-Day	Inter-Day		Mean ± SD	CV (%)
Plasma of pigs	PLP	LLOQ	5.00	14.10	−2.00	97.80 ± 4.76	4.87
		QCL	8.47	12.61	5.08	98.00 ± 7.75	7.91
		QCM	7.43	11.63	11.30	97.32 ± 7.22	7.42
		QCH	4.31	7.58	4.44	99.04 ± 4.30	4.34
	PA	LLOQ	10.56	15.49	6.60	95.31 ± 10.35	10.86
		QCL	4.41	11.22	5.70	98.37 ± 4.27	4.34
		QCM	9.42	8.12	11.91	95.78 ± 9.64	10.06
		QCH	3.04	4.59	1.80	99.87 ± 3.03	3.03
	PL	LLOQ	7.32	14.32	6.74	90.00 ± 7.07	7.86
		QCL	6.31	8.94	6.91	101.33 ± 6.39	6.31
		QCM	2.71	4.93	4.16	101.86 ± 2.76	2.71
		QCH	3.83	6.74	1.17	99.51 ± 3.81	3.83
Plasma of mice	PLP	LLOQ	9.03	15.55	15.38	90.19 ± 9.11	10.10
		QCL	6.41	12.56	3.74	98.00 ± 6.28	6.41
		QCM	6.17	13.30	3.63	91.67 ± 5.65	6.16
		QCH	3.40	8.73	2.01	95.68 ± 3.25	3.40
	PA	LLOQ	7.59	15.06	−7.30	89.00 ± 6.75	7.58
		QCL	7.46	7.58	−0.18	90.17 ± 6.73	7.46
		QCM	4.20	5.02	2.37	92.80 ± 3.90	4.20
		QCH	8.43	8.38	6.10	98.33 ± 8.29	8.43
	PL	LLOQ	4.81	17.33	−3.23	93.00 ± 4.47	4.81
		QCL	9.22	11.22	5.59	107.33 ± 9.90	9.22
		QCM	10.76	5.12	−4.65	113.90 ± 12.25	10.76
		QCH	4.01	7.44	−5.42	100.65 ± 4.04	4.01
Plasma of rats	PLP	LLOQ	7.89	15.39	1.06	94.00 ± 7.42	7.89
		QCL	10.34	12.41	6.50	92.33 ± 9.55	10.34
		QCM	6.21	10.47	0.31	106.33 ± 6.60	6.21
		QCH	8.37	3.86	5.74	101.76 ± 8.52	8.37
	PA	LLOQ	10.14	12.49	3.17	94.50 ± 9.59	10.15
		QCL	7.79	8.34	−2.72	94.23 ± 7.34	7.79
		QCM	6.26	7.92	9.70	94.80 ± 5.93	6.26
		QCH	10.63	5.87	6.49	95.53 ± 10.15	10.62
	PL	LLOQ	12.51	9.62	−7.61	92.00 ± 11.51	12.51
		QCL	6.63	6.96	−6.57	96.33 ± 6.39	6.63
		QCM	4.90	1.80	5.47	103.92 ± 5.10	4.91
		QCH	6.26	2.50	2.83	99.00 ± 6.20	6.26

**Table 8:** Short-term stability of PLP, PA, and PL in plasma of pigs, mice, and rats.

Compounds	QC (*n* = 6)	Plasma of Pigs		Plasma of Mice		Plasma of Rats	
		Concentration Found (nmol/L)	RSD (%)	Concentration Found (nmol/L)	RSD (%)	Concentration Found (nmol/L)	RSD (%)
PLP	QCL	0.59 ± 0.08	13.66	0.49 ± 0.02	4.33	0.51 ± 0.06	12.34
	QCM	1.29 ± 0.07	5.48	1.10 ± 0.07	11.49	1.29 ± 0.19	13.02
	QCH	5.10 ± 0.34	6.62	47.50 ± 3.59	10.13	49.84 ± 1.66	9.90
PA	QCL	1.09 ± 0.09	8.41	1.21 ± 0.14	6.65	1.29 ± 0.17	14.97
	QCM	2.63 ± 0.20	7.78	2.12 ± 0.28	13.37	2.32 ± 0.34	14.76
	QCH	10.02 ± 0.54	5.42	116.96 ± 3.04	6.98	118.36 ± 2.84	8.5
PL	QCL	59.20 ± 3.25	5.49	5.06 ± 0.51	7.55	5.13 ± 0.51	3.33
	QCM	130.19 ± 3.00	2.30	10.29 ± 0.72	2.60	10.14 ± 0.86	2.40
	QCH	518.70 ± 14.98	2.89	509.50 ± 18.11	3.56	486.92 ± 10.27	2.11

**Table 9:** Distribution of plasma PLP, PA, and PL in pigs, mice, and rats.

Plasma Sample	Compounds		
	PLP (nmol/L)	PA (nmol/L)	PL (nmol/L)
Pigs (*n* = 10)	197~431	3~15	82~185
Mice (*n* = 11)	131~256	10~15	25~130
Rats (*n* = 12)	151~300	9~51	21~36

## Data Availability

Date are contained within the article.

## Supplementary Materials

The following supporting information can be downloaded at: https://www.mdpi.com/article/10.3390/ani13081333/s1, Table S1: Recoveries of PLP, PA and PL extraction at different incubation times and temperatures; Table S2: Plasma matrix curves of pigs, mouse and rats.
