# Supplementary material for: A Novel Two-Dimensional Liquid Chromatography Combined with Ultraviolet Detection Method for Quantitative Determination of Pyridoxal 5′-Phosphate, 4-Pyridoxine Acid and Pyridoxal in Animal Plasma
Source: Animals (Basel). 2023 Apr 13;13(8):1333. doi: 10.3390/ani13081333 (PMC10135266; doi:10.3390/ani13081333)
Supplement: Supplementary file 1 [file animals-13-01333-s001.zip › animals-2134238-supplementary.pdf]

A novel two-dimensional liquid chromatography combined with ultraviolet detection method for quantitative determination of pyridoxal 5'-phosphate, 4-pyridoxine acid and pyridoxal in animal plasma.

Rong-Ju Yang [1,2,†], Na Wang [1,2,†], Xiao Ma [1,2], Meng-Die Gong [1,2], Yi-Rong Wang [1,2], Si-Yu Meng [1,2], Zhao-Ying Liu [1,2,*] and Qi Tang [3,*]

1 College of Veterinary Medicine, Hunan Agricultural University, Changsha 410128, China
2 Hunan Engineering Technology Research Center of Veterinary Drugs, Hunan Agricultural University, Changsha 410128, China
3 College of HNOorticulture, Hunan Agricultural University, Changsha 410128, China
* Correspondence: liu_zhaoying@hunau.edu.cn (Z.-Y.L.); tangqi@hunau.edu.cn (Q.T.)
† These authors contributed equally to this work.

Table S1. Recoveries of PLP, PA and PL extraction at different incubation times and temperatures

Incubation time	Incubation temperature														
	**0℃**			**4℃**			**20℃**			**37℃**			**50℃**		
	**PLP**	**PA**	**PL**	**PLP**	**PA**	**PL**	**PLP**	**PA**	**PL**	**PLP**	**PA**	**PL**	**PLP**	**PA**	**PL**
5 min	93%	97%	95%	96%	101%	98%	97%	100%	98%	99%	104%	101%	99%	102%	99%
10 min	96%	103%	95%	98%	101%	99%	95%	100%	89%	100%	104%	99%	97%	101%	97%
20min	96%	98%	96%	95%	98%	93%	112%	118%	113%	96%	100%	92%	91%	103%	83%
30 min	93%	93%	94%	86%	97%	86%	94%	97%	92%	96%	99%	90%	101%	104%	96%
60 min	88%	91%	91%	83%	88%	84%	93%	98%	89%	95%	98%	89%			

Table S2. Plasma matrix curves of pigs, mouse and rats

Dilution ratio	Surrogate matrix	Compounds	Linearity	R^2	Slope （RSD%）
Diluted 50 times	Plasma of pigs	PLP	y=320.35x+4429.6	1	3.60%
		PA	y=251.79x+6048.1	0.9998	12.67%
		PL	y=317.61x-1542.5	0.9999	3.33%
	Plasma of mice	PLP	y=313.16x+3629.7	0.9999	9.39%
		PA	y=261.88x+5718.3	0.9985	3.42%
		PL	y =316.06x+3051.5	1	11.16%
	Plasma of rats	PLP	y =292.7x+5061.4	0.999	7.57%
		PA	y=254.28x+5845.9	0.9994	11.61%
		PL	y=315.23x+1727.9	0.9999	7.18%
Diluted 100 times	Plasma of pigs	PLP	y=293.68x+4232.2	0.9994	5.12%
		PA	y=249.47x+4426.4	0.9997	12.72%
		PL	y=302.76x-1510.1	0.9991	4.54%
	Plasma of mice	PLP	y=306.82x+4697.4	0.9995	7.11%
		PA	y=246.05x+4014.4	0.9999	12.86%
		PL	y=301.63x-3590.1	0.9975	7.19%
	Plasma of rats	PLP	y =292.7x+5061.4	0.999	7.57%
		PA	y=254.28x+5845.9	0.9994	11.61%
		PL	y=315.23x+1727.9	0.9999	7.18%
